# ACD-DETR: Adaptive Cross-Scale Detection Transformer for Small Object Detection in UAV Imagery

**DOI:** 10.3390/s25175556

**Published:** 2025-09-05

**Authors:** Yang Tong, Hui Ye, Jishen Yang, Xiulong Yang

**Affiliations:** 1 School of Computer Science, Central China Normal University, Wuhan 430079, China; tongyang@mails.ccnu.edu.cn; 2 Department of Computer Science, Georgia State University, Atlanta, GA 30303, USA; hye2@student.gsu.edu; 3 Amazon, Seattle, WA 98109, USA; jishen.yang@hotmail.com; 4 Hubei Provincial Key Laboratory of Artificial Intelligence and Smart Learning, Central China Normal University, Wuhan 430079, China

**Keywords:** small object detection, Transformer, feature fusion, edge enhancement, positional encoding

## Abstract

Small object detection in UAV imagery remains challenging due to complex aerial perspectives and the presence of dense, small targets with blurred boundaries. To address these challenges, we propose ACD-DETR, an adaptive end-to-end Transformer detector tailored for UAV-based small object detection. The framework introduces three core modules: the Multi-Scale Edge-Enhanced Feature Fusion Module (MSEFM) to preserve fine-grained details; the Omni-Grained Boundary Calibrator (OG-BC) for boundary-aware semantic fusion; and the Dynamic Position Bias Attention-based Intra-scale Feature Interaction (DPB-AIFI) to enhance spatial reasoning. Furthermore, we introduce ACD-DETR-SBA+, a fusion-enhanced variant that removes OG-BC and DPB-AIFI while deploying densely connected Semantic–Boundary Aggregation (SBA) modules to intensify boundary–semantic fusion. This design sacrifices computational efficiency in exchange for higher detection precision, making it suitable for resource-rich deployment scenarios. On the VisDrone2019 dataset, ACD-DETR achieves 50.9% mAP@0.5, outperforming the RT-DETR-R18 baseline by 3.6 percentage points, while reducing parameters by 18.5%. ACD-DETR-SBA+ further improves accuracy to 52.0% mAP@0.5, demonstrating the benefit of SBA-based fusion. Extensive experiments on the VisDrone2019 and DOTA datasets demonstrate that ACD-DETR achieves a state-of-the-art trade-off between accuracy and efficiency, while ACD-DETR-SBA+ achieves further performance improvements at higher computational cost. Ablation studies and visual analyses validate the effectiveness of the proposed modules and design strategies.

## 1. Introduction

As one of the important tasks in the field of computer vision, object detection aims to identify and locate objects of specific categories in an image. It is widely used in security monitoring, autonomous driving, smart cities, and other scenarios.

In recent years, with the rapid development of deep learning technology, object detection algorithms have achieved significant improvements in accuracy and efficiency. However, when applied to UAV platforms, traditional object detection methods face severe challenges due to highly flexible flight platforms and complex, variable imaging environments.

UAVs offer high mobility, broad coverage, and low deployment cost, making them suitable for aerial monitoring, environmental sensing, and disaster response. However, UAV imagery often suffers from low resolution, large variations in object scale, unique aerial viewpoints, and complex occlusion and lighting conditions, which seriously affect the generalization ability and detection accuracy of object detection models. Especially in small object detection scenarios, traditional methods struggle to effectively identify and localize small objects due to their limited semantic information, small size, and blurred boundaries.

As the first end-to-end model based on the Transformer architecture, DETR [1] has attracted widespread attention due to its novel design. However, its high computational cost and poor real-time performance make it unsuitable for real-time scenarios. To address this, RT-DETR [2] significantly improves inference efficiency while maintaining detection accuracy, providing an effective solution for real-time object detection. Nevertheless, existing methods still face key limitations in dense small object scenes: (1) the convolutional feature extractor has limited ability to retain edge and detail information; (2) semantic inconsistencies and boundary weakening occur during multi-scale feature fusion; and (3) fixed positional embeddings cannot effectively model complex spatial relationships, limiting the spatial modeling capability of the model.

To overcome these limitations, we propose ACD-DETR (Adaptive Cross-scale Detection Transformer), a novel and lightweight framework specifically designed for robust and efficient detection of small objects. The architecture introduces three core modules to enhance spatial sensitivity, semantic consistency, and positional modeling capability.

Our main contributions are summarized as follows:

1. We propose a Multi-Scale Edge-Enhanced Feature Fusion Module (MSEFM) that combines multi-scale pooling, explicit edge feature enhancement, and channel attention to enrich fine-grained feature representations, especially for small objects across scales (i.e., integrating features from different spatial resolutions to combine detail-rich shallow features with semantically strong deep features).
2. We design a novel Omni-Grained Boundary Calibrator (OG-BC) that hierarchically fuses semantic and structural information, utilizing dynamic alignment and boundary-aware interaction to enhance object localization in dense scenes.
3. We introduce a Dynamic Position Bias Attention-based Intra-scale Feature Interaction (DPB-AIFI) model to replace fixed positional embeddings with learnable, relative spatial priors, thereby improving spatial modeling and enhancing detection accuracy under complex object layouts.

The remainder of this paper is organized as follows. Section 2 reviews related work on UAV object detection and real-time end-to-end detectors. Section 3 details the proposed ACD-DETR framework, including the MSEFM, OG-BC, and DPB-AIFI modules. Section 4 presents the experimental setup, evaluation results, and ablation studies. Finally, Section 5 concludes the paper and discusses future work.

## 2. Related Work

### 2.1. Object Detection in UAV Imagery

Object detection algorithms are generally categorized into two-stage and one-stage methods. Two-stage detectors, such as the Faster R-CNN series [3] and its variants, first generate candidate regions via a Region Proposal Network (RPN) and then perform feature extraction for classification and bounding box regression, typically achieving higher detection accuracy in complex scenarios. Building on this paradigm, Magoulianitis et al. introduced a super-resolution reconstruction module to improve image detail and recall rate [4]. Lin et al. proposed the Feature Pyramid Network (FPN) [5], which enhanced multi-scale feature representation through lateral connections, effectively addressing the issue of rich semantics but poor detail in deep layers. Additionally, Cai et al. introduced Cascade R-CNN [6], which progressively increased the Intersection over Union (IoU) threshold to achieve more accurate localization.

While two-stage methods achieve high accuracy, their computational overhead limits real-time deployment on resource-constrained UAV platforms.This limitation motivated the community to shift toward one-stage detectors. The YOLO series [7,8,9,10,11] stands out as a representative framework, continuously improving small-object detection performance through anchor-based mechanisms, multi-scale feature maps, and lightweight modules. Wang et al. proposed YOLOX-w [12], which enhanced detection capabilities by incorporating shallow features, quantized attention modules, and data augmentation strategies. Li et al. introduced Bi-PAN-FPN and GhostBlockV2 into the YOLOv8 framework [13], achieving better feature transmission stability and representation while maintaining lightweight model complexity.

### 2.2. Real-Time End-to-End Object Detection

YOLOv1 [7] was the first end-to-end object detection framework based on Convolutional Neural Networks (CNNs), achieving real-time detection by dividing images into fixed grids and simultaneously predicting object categories and bounding boxes. Owing to its simple structure and fast inference speed, it demonstrated strong applicability in real-world scenarios. However, YOLOv1 and its successors relied heavily on Non-Maximum Suppression (NMS) during the post-processing stage to eliminate redundant candidate boxes. This introduced additional computational overhead and made the detection results highly sensitive to threshold settings, thereby compromising model robustness.

To overcome these limitations, Carion et al. proposed DETR [1], which was the first to introduce the Transformer architecture into object detection tasks. Based on sequence modeling, DETR leveraged self-attention mechanisms to globally model object relationships in the image, completely discarding the use of NMS and manually designed anchor boxes. This significantly simplified the detection pipeline and enhanced contextual understanding. However, the original DETR suffered from slow convergence during training and exhibited suboptimal performance in small object detection, limiting its applicability on resource-constrained platforms such as UAVs.

Following DETR, Fang et al. introduced YOLOS [14], a minimalist end-to-end detector built entirely on the Vision Transformer (ViT) architecture. YOLOS discards convolutional backbones and handcrafted multi-scale feature fusion, instead directly processing patch embeddings with Transformer encoders and predicting object queries in a DETR-like fashion. While this design achieves architectural simplicity and validates the feasibility of pure Transformer-based detection, the absence of explicit multi-scale feature aggregation limits its performance on small objects, making it less suitable for UAV scenarios where tiny targets are prevalent.

Given the dual requirements of real-time inference and high accuracy in UAV-based object detection, many researchers have turned to one-stage detectors represented by the YOLO series. These models maintain fast inference speeds while achieving satisfactory detection accuracy. Nonetheless, they still rely on NMS, making them sensitive to threshold parameters and potentially unstable during deployment.

To address this, Zhao et al. introduced RT-DETR [2], the first Transformer-based detector to achieve real-time inference in an end-to-end fashion. RT-DETR integrated cross-scale attention interaction modules, CNN-based feature fusion structures, and dynamic query selection mechanisms to eliminate the need for NMS, thereby achieving a better balance between accuracy and speed. Its global attention mechanism efficiently captured long-range dependencies and contextual information. Moreover, the lightweight design of its inference architecture significantly reduced computational cost, making it highly suitable for deployment in UAV scenarios with limited onboard resources.

## 3. Methodology

**Figure 1:**
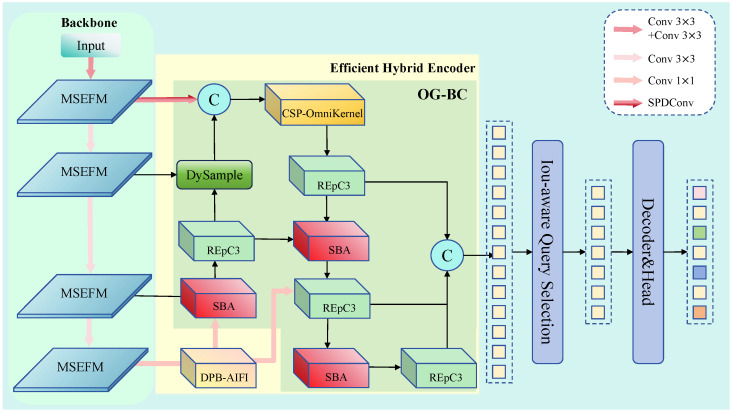
ACD-DETR structure.

This section presents ACD-DETR, an efficient end-to-end detection framework specifically designed for UAV-based small object detection. Figure 1 provides a comprehensive overview of the proposed architecture, which features multiple interconnected components designed to work collaboratively for enhanced small-object detection performance.

The overall architecture builds upon RT-DETR but incorporates three key innovations strategically positioned throughout the network to address fundamental small-object detection challenges. The framework follows a detection pipeline of feature extraction, feature fusion, and detection head stages, with significant enhancements improving small object perception and localization accuracy.

As shown in the left side of Figure 1, the MSEFM is integrated into each backbone stage. This module processes input features through multiple parallel branches with adaptive pooling scales (3 × 3, 6 × 6, 9 × 9, and 12 × 12), capturing contextual information at various scales. Each branch incorporates edge enhancement mechanisms that explicitly compensate for fine-grained details lost during downsampling, with outputs fused through an EMA (Efficient Multi-scale Attention) [15] attention mechanism to generate boundary-aware multi-scale representations.

The middle portion illustrates the OG-BC, which replaces traditional feature pyramid structures with intelligent fusion. Rather than using P2 features as direct detection heads, OG-BC strategically employs them as guidance through SPDConv [16] operations while maintaining computational efficiency. The hierarchical fusion pathway alternately applies SBA and RepC3 (Reparameterized C3) blocks, with DySample (Upsampling by Dynamic Sampling) [17] modules ensuring adaptive multi-scale semantic alignment.

The right side shows the enhanced encoder where traditional AIFI modules are replaced with DPB-AIFI. This introduces learnable dynamic position bias through Multi-Layer Perceptron networks, enabling adaptive modeling of the spatial relationships crucial for complex aerial imagery layouts.

Together, these modules create a comprehensive small-object detection capability by addressing fundamental challenges. MSEFM mitigates information loss during downsampling by preserving fine-grained details across multiple receptive field scales. It also explicitly enhances boundary information, which is critical for small object discrimination. OG-BC resolves semantic inconsistency across feature pyramid levels through guided fusion mechanisms, maintaining scale-consistent representations without the semantic dilution typical of naive concatenation approaches. DPB-AIFI models complex spatial dependencies in dense aerial scenes, with learnable position bias improving small object discrimination from background clutter and handling occlusions where static positional encodings prove insufficient.

Furthermore, the integrated design reduces parameters while improving detection accuracy, demonstrating that architectural innovations can achieve both effectiveness and efficiency for real-time applications.

### 3.1. Multi-Scale Edge-Enhanced Feature Fusion Module

In general object detection tasks, small objects are inherently challenging due to their limited size, blurred boundaries, and weak semantic information, making them difficult to model effectively with deep networks. In particular, repeated convolution and downsampling operations tend to weaken or even eliminate the fine-grained edges and high-frequency details originally present in the image, leading to incomplete feature representations and frequent instances of missed or false detections.

Moreover, small object detection is highly sensitive to contextual information. Unlike large objects that occupy more spatial area, small objects rely more heavily on multi-scale semantic cues to provide discriminative features. However, traditional feature extraction networks often lack the ability to perceive edge details, and their fixed-scale convolutional structures are insufficient for capturing rich multi-scale contextual information. Therefore, it is essential to develop a lightweight feature extraction module that can simultaneously enhance edge representation and contextual modeling, thereby improving the backbone network’s perception of complex small objects.

To address this, we propose a novel feature extraction module called the Multi-Scale Edge-Enhanced Feature Fusion Module (MSEFM). This module is designed with a synergistic approach that combines multi-scale receptive field expansion, explicit edge enhancement, and channel-wise attention mechanisms. It dynamically reconstructs the input features at each stage of the backbone network, significantly improving the network’s edge awareness and modeling capability for small objects.

**Figure 2:**
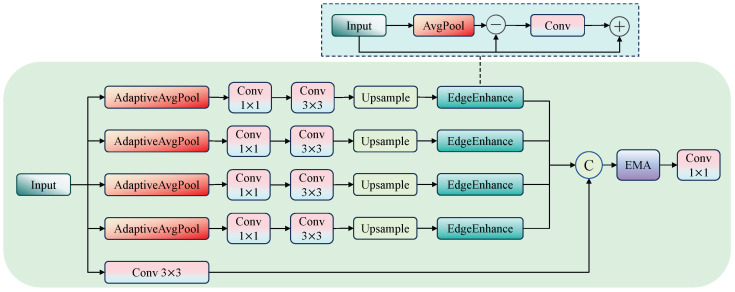
Multi-Scale Edge-Enhanced Feature Fusion Module structure.

As shown in Figure 2, the module first applies multi-scale adaptive average pooling (e.g., bins = [3, 6, 9, 12]) to the input features to construct multiple scale branches, enabling the extraction of both local and global contextual information. The multi-scale feature extraction process is defined as:

(1) $$F_{i}^{\prime }={\mathrm{Conv}}_{3\times 3}\left({\mathrm{Conv}}_{1\times 1}\left({\mathrm{AdaptiveAvgPool}2d}_{{\mathrm{bin}}_{i}}\left(X\right)\right)\right),\;i\in \left\{\mathrm{bins}\right\}$$

Here, i∈{bins} and *X* denotes the input feature map. Each scale branch applies a 1×1 convolution to reduce the channel dimensionality, followed by a 3×3 convolution to capture spatial structural information. This process effectively expands the receptive field to support multi-scale target modeling.

Next, an edge enhancement module is embedded in each scale branch. The edge enhancement mechanism is formulated as

(2) $$F_{\mathrm{edge}}=F-{\mathrm{AvgPool}}_{3\times 3}\left(F\right)$$

(3) $$F_{\mathrm{enhanced}}=F+F⊙\sigma \left(\mathrm{Conv}(F_{\mathrm{edge}})\right)$$

Here, σ denotes the sigmoid activation function and ⊙ represents element-wise multiplication. The module first extracts a smoothed version of the input feature using average pooling, computes the edge response map by subtracting it from the original feature, and then generates an edge attention map through convolution followed by sigmoid activation. This attention map is used to weight the original feature, effectively enhancing fine-grained details. The mechanism explicitly compensates for edge information lost during downsampling and makes the network more sensitive to contour variations, which is especially beneficial for detecting dense small objects.

Finally, all enhanced features from different scales are upsampled and concatenated along the channel dimension to form the fused feature map. To further emphasize key semantics and suppress redundant information, an EMA module is introduced. EMA divides the feature channels into groups and uses multi-branch pathways to capture global context and spatial dependencies, dynamically generating attention weights to filter information across channels and spatial locations.

The complete computation flow of the MSEFM module can be summarized as

(4) $$F_{\mathrm{local}}={\mathrm{Conv}}_{3\times 3}\left(X\right)$$

(5) $$F_{\mathrm{multi}}=\left\{{\mathrm{EE}}_{i}\left(\mathrm{Upsample}\left(F_{i}^{\prime }\right)\right)\;|\;i\in \mathrm{bins}\right\}$$

(6) $$F_{\mathrm{output}}={\mathrm{Conv}}_{1\times 1}\left(\mathrm{EMA}\left(\mathrm{Concat}\left(F_{\mathrm{local}}\|F_{\mathrm{multi}}\right)\right)\right)$$

where ${\mathrm{EE}}_{i}$ denotes the edge enhancement module in the *i*-th scale branch, calculated according to the edge enhancement equations above. Finally, a 1×1 convolution is applied to integrate all channels and produce high-quality fused features.

### 3.2. Omni-Grained Boundary Calibrator

In dense-small-object detection tasks, conventional detection layers such as P3, P4, and P5 often struggle to adequately capture the boundaries and semantic details of tiny objects. Although directly introducing a P2 detection head can partially alleviate this issue, it significantly increases the model’s computational overhead and post-processing complexity. Traditional approaches that use P2 features as an additional detection head require extra prediction layers, anchor processing, and post-processing mechanisms, substantially increasing computational cost. To address this trade-off, we propose the Omni-Grained Boundary Calibrator (OG-BC) module, a feature fusion architecture that balances small object perception capability with computational efficiency.

The fundamental challenge lies in the inherent contradiction between shallow and deep features: shallow features possess rich spatial details and clear boundaries but lack semantic context, while deep features contain abundant semantic information but suffer from spatial resolution loss. Direct fusion often leads to semantic inconsistency and information redundancy, which is particularly problematic for tiny objects requiring both precise localization and semantic understanding.

**Figure 3:**
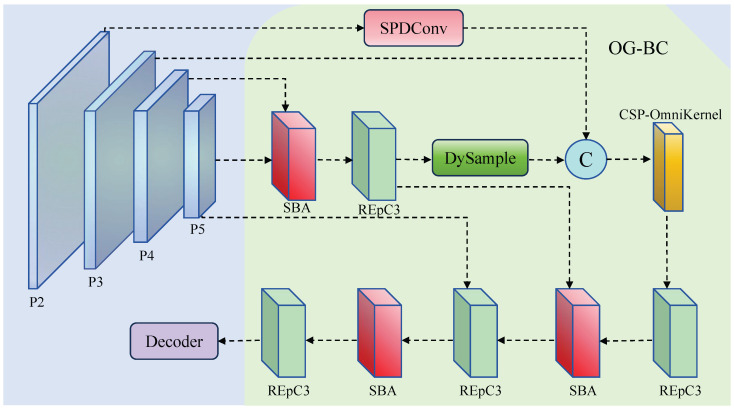
Omni-Grained Boundary Calibrator structure.

As illustrated in Figure 3, the overall architecture of the OG-BC module adopts a hierarchical fusion strategy. Crucially, unlike traditional methods that create an additional P2 detection head, our approach uses P2 features as guidance information only. Specifically, the P2 feature map is utilized as a key guidance source but is no longer directly used as a detection head. Instead, a lightweight SPDConv [16] operation is employed to extract spatial fine-grained structures and small object information from P2, which are then deeply fused into the P3 layer. This design maintains the same number of detection heads as the baseline while leveraging P2’s spatial richness, achieving enhanced perception capability without computational overhead of additional detection processing.

To further enhance feature consistency and expressiveness, OG-BC integrates a DySample [17] module based on dynamic weight adjustment, enabling adaptive alignment of multi-scale semantics and effectively addressing the scale and semantic misalignment issues commonly found in feature pyramids.

Along the feature fusion pathway, OG-BC alternately employs the SBA and RepC3 modules to construct refined network blocks, thereby enhancing the network’s sensitivity to local boundaries and scale variations. Finally, the fused features are fed into an improved CSP-OmniKernel module for high-level semantic modeling. Through this design, OG-BC achieves explicit modeling and boundary enhancement for small objects, significantly improving detection accuracy and localization robustness for tiny objects without introducing additional detection heads.

#### 3.2.1. CSP-OmniKernel

**Figure 4:**
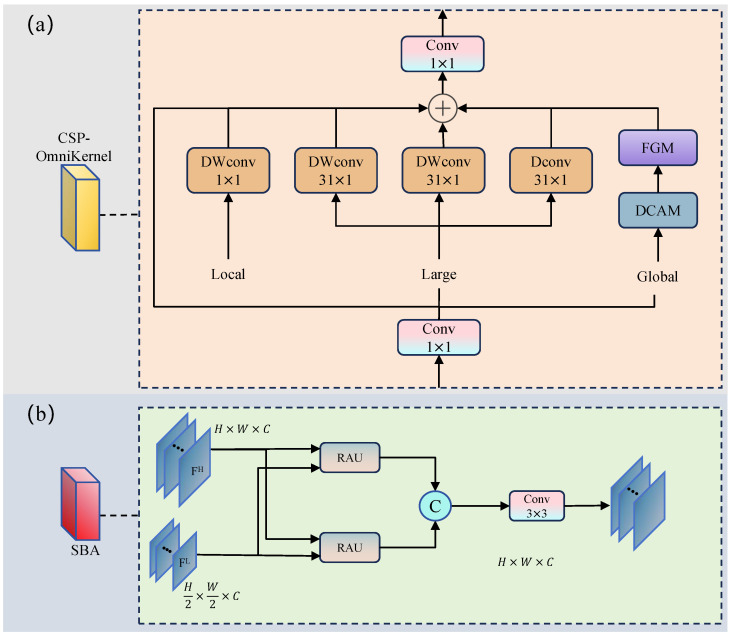
(**a**) CSP-OmniKernel module structure, which combines the channel splitting strategy of CSP architecture with OmniKernel’s multi-domain feature modeling capability. (**b**) Semantic–Boundary Aggregation (SBA) module structure, designed to guide bidirectional interaction and reconstruction between shallow boundary information and deep semantic features.

To effectively exploit large-kernel convolution while maintaining computational efficiency, we propose the CSP-OmniKernel module, which integrates the channel splitting strategy of the Cross Stage Partial (CSP) architecture with the multi-domain feature modeling capability of OmniKernel [18]. As illustrated in Figure 4a, the CSP-OmniKernel adopts a branch-and-fusion design that balances representation ability and computational overhead.

Given an input feature map $X\in R^{C\times H\times W}$, the input is first projected via a 1×1 convolution and then split along the channel dimension:

(7) $$X^{\prime }={\mathrm{Conv}}_{1\times 1}\left(X\right),\;X_{\mathrm{Omni}},X_{\mathrm{ID}}=\mathrm{Split}\left(X^{\prime },e\right)$$

where *e* denotes the channel split ratio (set to e=0.25 by default). The smaller portion $X_{\mathrm{Omni}}$ is processed through the OmniKernel branch, while the larger portion $X_{\mathrm{ID}}$ undergoes identity mapping to preserve computational efficiency.

In the OmniKernel branch, multi-branch depthwise convolutions with different receptive fields are applied in parallel. Their outputs are fused via element-wise addition and combined with a dual-domain attention-enhanced residual connection:

(8) $$\begin{array}{cc} X_{\mathrm{kernel}}=X_{\mathrm{pre}} & +{\mathrm{DWConv}}_{1\times k}\left(X_{\mathrm{pre}}\right)+{\mathrm{DWConv}}_{k\times 1}\left(X_{\mathrm{pre}}\right)\\ \; & +{\mathrm{DWConv}}_{k\times k}\left(X_{\mathrm{pre}}\right)+{\mathrm{DWConv}}_{1\times 1}\left(X_{\mathrm{pre}}\right)+F_{\mathrm{att}} \end{array}$$

where $X_{\mathrm{pre}}$ is obtained through a 1×1 convolution followed by GELU activation and *k* denotes the large kernel size (set to k=31 by default).

The attention term $F_{\mathrm{att}}$ combines frequency-domain and spatial-domain enhancements using a dual-domain channel attention mechanism:

(9) $$F_{\mathrm{att}}=\mathrm{FGM}\left(\mathrm{SCA}(F^{-1}\left(\mathrm{FCA}\left(F\left(X_{\mathrm{pre}}\right)\right)\right))\right)$$

where F(·) and $F^{-1}\left(\cdot \right)$ denote the Fourier transform and its inverse, respectively; FCA(·) and SCA(·) represent frequency channel attention and spatial channel attention modules. The combination of FCA and SCA constitutes the Dual-Domain Channel Attention Module (DCAM), which captures complementary frequency and spatial features to enhance channel representations. FGM(·) is a frequency-enhanced gating module that further refines the attended features.

Finally, the outputs from the OmniKernel branch and the identity-mapped features are concatenated and projected to produce the final output via a 1×1 convolution:

(10) $$Y={\mathrm{Conv}}_{1\times 1}\left(\mathrm{Concat}(X_{\mathrm{kernel}},X_{\mathrm{ID}})\right)$$

The proposed CSP-OmniKernel module effectively models multi-scale spatial structures and captures global semantic information while maintaining low computational cost, thereby contributing to robust feature extraction for small-object detection tasks.

#### 3.2.2. Semantic–Boundary Aggregation

In visual feature pyramids, shallow features typically possess higher spatial resolution and superior detail representation capabilities, particularly excelling in boundary localization and shape depiction. Conversely, deep features contain richer semantic context that aids in object classification and structural recognition. However, the disparities in scale and semantic abstraction between these feature types can introduce redundancy or semantic inconsistency when directly fused, thereby limiting the representational effectiveness and detection performance of multi-scale fusion.

To address these challenges, we propose the Semantic–Boundary Aggregation (SBA) module, which achieves precise object representation through bidirectional gated fusion between shallow boundary information and deep semantic features. The SBA module enhances feature complementarity while maintaining computational efficiency through simple yet effective operations.

As illustrated in Figure 4b, the SBA module takes two scales of feature maps as input: high-resolution boundary features $F^{H}\in R^{C_{H}\times H\times W}$ and low-resolution semantic features $F^{L}\in R^{C_{L}\times \frac{H}{2}\times \frac{W}{2}}$. Both feature maps are first projected through 1×1 convolutions for channel alignment, with attention gates simultaneously generated via sigmoid activations:

(11) $$F_{c}^{H}={\mathrm{Conv}}_{1\times 1}^{H}\left(F^{H}\right),\;F_{c}^{L}={\mathrm{Conv}}_{1\times 1}^{L}\left(F^{L}\right)$$

(12) $$g_{H}=\sigma \left(F_{c}^{H}\right),\;g_{L}=\sigma \left(F_{c}^{L}\right)$$

These gates adaptively regulate the contribution of features at different resolutions during the fusion process.

The projected features are further processed through lightweight convolutions to enhance their representational capacity:

(13) $${\tilde{F}}_{c}^{H}={\mathrm{Conv}}_{1\times 1}^{proc}\left(F_{c}^{H}\right),\;{\tilde{F}}_{c}^{L}={\mathrm{Conv}}_{1\times 1}^{proc}\left(F_{c}^{L}\right)$$

The core of SBA lies in the Re-calibration Attention Unit (RAU), which performs bidirectional gated fusion through a three-term formulation. We define RAU as

(14) $$\mathrm{RAU}\left(X,Y,g_{X},g_{Y}\right)=X+X⊙g_{X}+\left(1-g_{X}\right)⊙\mathrm{Upsample}\left(g_{Y}⊙Y\right)$$

where *X* is the target feature, *Y* is the source feature, and $g_{X}$ and $g_{Y}$ are the corresponding gates. The enhanced features are computed as

(15) $${\mathrm{RAU}}^{L}=\mathrm{RAU}\left({\tilde{F}}_{c}^{L},{\tilde{F}}_{c}^{H},g_{L},g_{H}\right),\;{\mathrm{RAU}}^{H}=\mathrm{RAU}\left({\tilde{F}}_{c}^{H},{\tilde{F}}_{c}^{L},g_{H},g_{L}\right)$$

The RAU design incorporates three essential components: (1) residual connection for gradient flow preservation, (2) self-gating for adaptive feature selection, and (3) cross-branch integration for complementary information enhancement. This mechanism ensures that each branch preserves its intrinsic characteristics while adaptively incorporating beneficial information from the complementary branch through a learnable gating strategy.

Finally, the enhanced high-resolution features are spatially aligned to match the low-resolution scale, and both branches are concatenated for final fusion:

(16) $$F_{\mathrm{SBA}}={\mathrm{Conv}}_{3\times 3}\left(\mathrm{Concat}\left(\mathrm{Upsample}\left({\mathrm{RAU}}^{H}\right),{\mathrm{RAU}}^{L}\right)\right)$$

Compared to traditional FPN structures, the SBA module offers several key advantages. First, the RAU-based gated fusion mechanism effectively balances self-enhancement and cross-branch information exchange, reducing redundancy while preserving complementary features. Second, the lightweight design incurs minimal computational overhead through simple convolution and gating operations, making it suitable for resource-constrained scenarios. Third, the bidirectional enhancement strategy enables superior multi-scale feature integration, which is particularly beneficial for small-object detection tasks that require both detailed boundary information and rich semantic context.

The proposed SBA module demonstrates that effective multi-scale fusion can be achieved through carefully designed gating mechanisms without complex attention computations, thereby offering a practical solution for real-time applications while maintaining strong representational capability.

### 3.3. DPB-AIFI

In RT-DETR, the traditional AIFI module processes the relationships between different positions in the input feature sequence through the Multi-Head Attention (MSA) mechanism. MSA divides the input sequence into multiple attention heads, allowing each head to independently perform self-attention calculations, thereby enabling efficient parallel processing of complex dependencies within the sequence. However, the fixed positional encoding used in traditional self-attention mechanisms has significant limitations. Its predefined positional representations struggle to adapt to input features of varying scales and has limited capability for modeling relative positional relationships in continuous space, which affects the model’s ability to accurately capture critical spatial information.

To address these issues, we introduce a dynamic position bias (DPB) [19] mechanism in the feature interaction module, constructing the DPB-AIFI module. This module dynamically generates positional encodings that adapt to the scale of the input features, effectively enhancing the model’s ability to perceive spatial relationships at any scale, reducing information loss caused by fixed positional encodings, and improving the understanding of spatial structures in object detection tasks.

**Figure 5:**
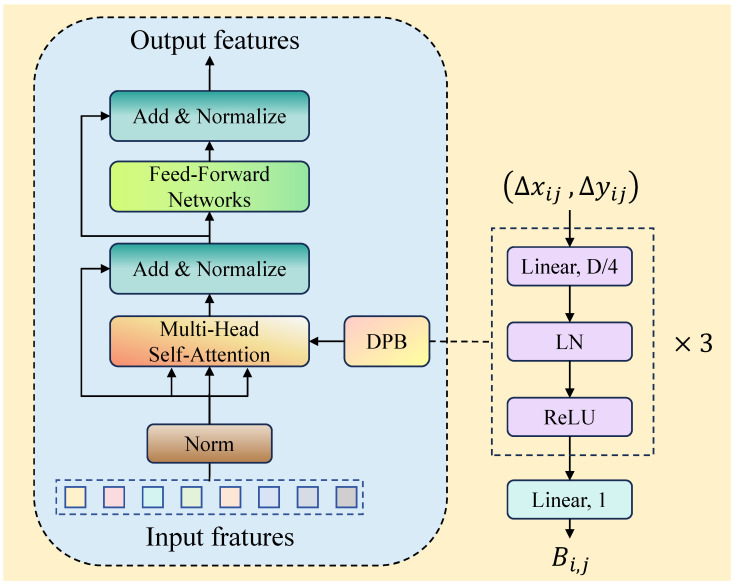
DPB-AIFI structure.

As shown in Figure 5, the core innovation of the DPB-AIFI module lies in dynamically generating positional biases through a Multi-Layer Perceptron (MLP). The dynamic position bias generation process can be expressed as

(17) $$B_{i,j}=DPB\left(\Delta x_{ij},\Delta y_{ij}\right)$$

where (DPB) represents the dynamic position bias generation function and ($\Delta x_{ij},\Delta y_{ij}$) denotes the relative coordinate distance between position i and position j.

Specifically, this process first inputs the relative positional coordinates into an MLP composed of three fully connected layers, each containing linear transformations, layer normalization, and ReLU activation functions. The first layer maps the input 2D relative coordinates to a hidden space of D/4 dimensions, followed by nonlinear transformations through layer normalization and ReLU activation, ultimately outputting a one-dimensional positional bias value. This progressive feature transformation effectively captures the complex nonlinear spatial relationships between different positions in the input feature map, ensuring that the generated positional biases contain rich spatial structural information.

The introduction of dynamic position bias significantly improves the computation process of traditional attention mechanisms. In the attention enhancement calculation, the module adds the dynamically generated positional bias $B_{i,j}$ to the traditional query–key dot-product attention scores, forming the enhanced attention calculation formula

(18) $$\mathrm{Attention}=\mathrm{Softmax}\left(\frac{QK^{T}}{\sqrt{d}}+B\right)V$$

This design allows each positional pair to obtain personalized positional weights, enabling the model to dynamically adjust the attention distribution based on specific spatial relationships. During computation, the dynamic position bias is added to the query–key dot-product scores and normalized through Softmax to generate the final attention weight distribution. In this way, DPB-AIFI not only retains the global modeling capability of traditional multi-head self-attention mechanisms but also enhances precise perception of local spatial structures, demonstrating stronger adaptability in object detection scenarios with complex spatial layouts.

Compared to traditional fixed positional encoding schemes, the dynamic position bias mechanism offers superior generalization and representational capabilities. Traditional methods often rely on predefined sinusoidal or cosine positional encodings, which struggle to adapt to input features of varying scales and resolutions. In contrast, the DPB mechanism uses a learnable parametric network to adaptively generate optimal positional representations based on specific tasks and data characteristics. This continuous relative positional modeling eliminates dependence on input scale and enables more precise capture of spatial relationships between any two positions, providing more accurate spatial priors for subsequent feature interactions and object detection.

In summary, the DPB-AIFI module integrates dynamic position bias with multi-head self-attention mechanisms to construct a feature interaction architecture that can precisely model positional relationships while capturing global semantic dependencies. This module significantly enhances RT-DETR’s understanding of complex spatial structures while maintaining computational efficiency, providing effective technical support for challenging visual tasks such as dense small object detection.

## 4. Experiments

### 4.1. Experimental Setup

The hardware and software configurations used during the experiments are detailed in Table 1. All experiments were conducted under the same settings to ensure consistency in the experimental environment. The hardware setup includes an AMD EPYC 7742 64-Core Processor (Advanced Micro Devices, Inc., Santa Clara, CA, USA) and an NVIDIA A100-SXM4-80 GB GPU (NVIDIA Corporation, Santa Clara, CA, USA), while the software environment consists of Ubuntu 22.04.5 LTS, Python 3.10.17, CUDA 11.8, and PyTorch 2.3.1.

To optimize training efficiency and model performance, the training parameters were adjusted following the official best practices of the RT-DETR model. The core training parameters are listed in Table 2, while other parameters were kept at their default values.

### 4.2. Datasets

**Figure 6:**
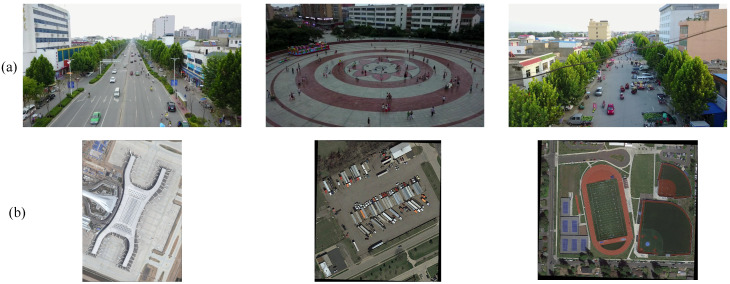
Example images from the datasets. (**a**) VisDrone2019 dataset. (**b**) DOTA dataset.

This study evaluates the ACD-DETR model using two representative remote sensing image datasets: VisDrone [20] and DOTA [21]. VisDrone serves as the primary experimental dataset, while DOTA is employed as a supplementary dataset to further assess the applicability of our method on a different aerial detection benchmark. Figure 6 shows representative examples from both datasets, illustrating the challenging nature of small object detection in aerial imagery.

**Figure 7:**
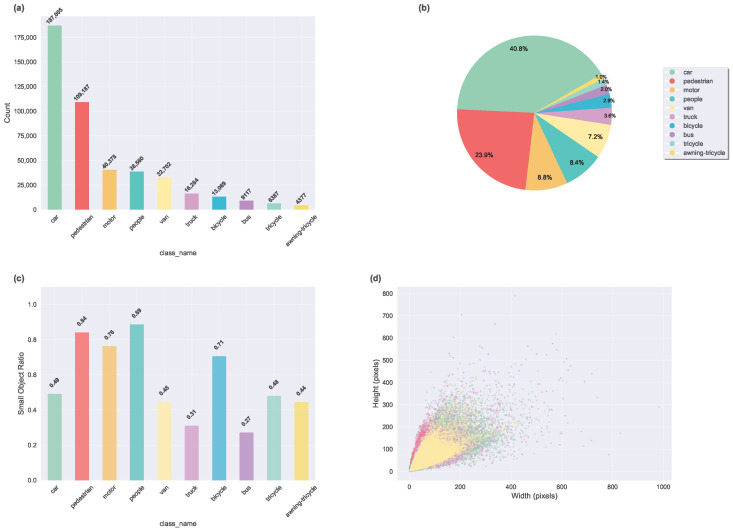
Statistical analysis of the VisDrone2019 dataset. (**a**) Class frequency distribution. (**b**) Category proportions. (**c**) Ratio of small objects (area <32×32 pixels) by class. (**d**) Object size distribution in terms of bounding-box width and height.

VisDrone is a large-scale UAV aerial image dataset constructed by the Machine Learning and Data Mining Laboratory at Tianjin University. It contains over 10,000 high-resolution images, covering diverse geographic locations, weather conditions, lighting environments, and shooting altitudes. The dataset is divided into a training set (6471 images), a validation set (548 images), and a test set (1610 images). It defines 10 target categories: pedestrian, people, bicycle, car, van, truck, tricycle, awning-tricycle, bus, and motor. The image resolutions range from 960×540 to 2000×1500 pixels, reflecting the diversity of UAV shooting conditions. Figure 7 provides a unified statistical analysis of the VisDrone2019 dataset, including class frequency distribution, category proportions, the ratio of small objects (area <32×32 pixels) [22] by class, and bounding-box size distributions.

**Figure 8:**
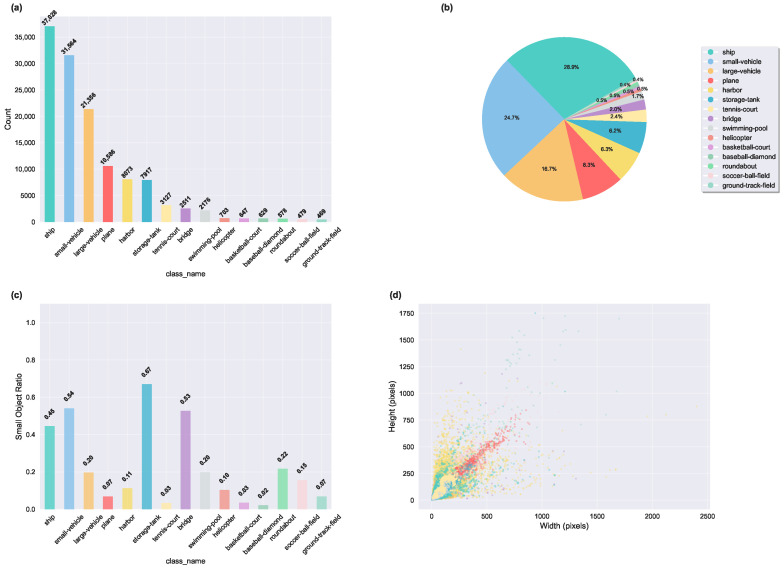
Statistical analysis of the DOTA dataset. (**a**) Class frequency distribution. (**b**) Category proportions. (**c**) Ratio of small objects (area <32×32 pixels) by class. (**d**) Object size distribution in terms of bounding-box width and height.

DOTA is a large-scale aerial image object detection dataset jointly released by Huazhong University of Science and Technology and other institutions. It contains 2806 large-scale aerial images with resolutions ranging from 800×800 to 4000×4000 pixels. The dataset defines 15 target categories: plane, ship, storage-tank, baseball-diamond, tennis-court, basketball-court, ground-track-field, harbor, bridge, large-vehicle, small-vehicle, helicopter, roundabout, soccer-ball-field, and swimming-pool. To process the large and varying-sized original images, we applied a sliding window method to crop them into 1024×1024 pixel sub-images with a 200-pixel overlap. After preprocessing, a total of 21,046 sub-images were generated, divided into 15,749 training images and 5297 test images. A statistical analysis of the dataset is presented in Figure 8, including class frequency distribution, object proportions, the ratio of small objects, and bounding-box size distributions.

### 4.3. Evaluation Metrics

To validate the effectiveness of the proposed algorithm, we selected several evaluation metrics, including precision, recall, average precision (AP), and mean average precision (mAP). These metrics were used to compare and analyze the algorithm’s performance.

In object detection tasks, TP, FP, and FN denote the numbers of true positive, false positive, and false negative detections, respectively, while TN (true negatives, where negative samples are correctly predicted as negative) is typically not considered due to the vast number of possible negative locations. A detection is counted as a true positive (TP) if it has the same class label as a ground-truth object and the Intersection over Union (IoU) between the predicted bounding box and the ground truth exceeds a threshold τ (0.5 for mAP@0.5, 0.75 for mAP@0.75), following the standard PASCAL VOC/COCO matching protocol. Each ground-truth object is matched with at most one detection; unmatched predictions are counted as false positives (FP), and unmatched ground-truth objects are counted as false negatives (FN). During evaluation, detections are ranked by their confidence scores, and a confidence threshold of 0.001 is applied to filter low-confidence predictions before IoU-based matching.

Precision measures the accuracy of the algorithm’s predictions, representing the proportion of correctly predicted positive samples among all predicted positive samples. It is calculated as

(19) $$\mathrm{Precision}=\frac{\mathrm{TP}}{\mathrm{TP}+\mathrm{FP}}$$

Recall measures the sensitivity of the algorithm, representing the proportion of correctly predicted positive samples among all actual positive samples. It is calculated as

(20) $$\mathrm{Recall}=\frac{\mathrm{TP}}{\mathrm{TP}+\mathrm{FN}}$$

AP evaluates the detection performance for a single category, while mAP is a comprehensive metric for evaluating the overall performance of object detection. mAP is calculated by averaging the AP values across all categories, as shown in the formula below:

(21) $$mAP=\frac{\sum AP}{NC}\times 100\%$$

where NC represents the number of categories and ∑AP is the sum of the average precision values for all categories.

Additionally, GFLOPs were used to measure computational complexity, and the number of parameters was used to reflect the model size.

### 4.4. Comparative Experiments

To evaluate the effectiveness of the proposed ACD-DETR, we conducted extensive comparative experiments on two challenging UAV datasets: VisDrone2019 and DOTA. All compared models were trained using identical configurations and hyperparameters to ensure a fair comparison. For each model and dataset combination, we performed three independent training runs with different random seeds, and the reported results represent the average performance across these runs to ensure statistical robustness.

Table 3 reports a detailed comparison of ACD-DETR with recent DETR variants on the VisDrone2019 dataset. ACD-DETR achieves 50.9% mAP@0.5, outperforming all compared DETR-based detectors while demonstrating significantly improved model efficiency. Specifically, it requires only 16.2M parameters, representing a 54.5% to 57.4% reduction compared to existing methods, and operates at 68.9 GFLOPs, which is substantially lower than Dynamic-DETR (85.0 GFLOPs) and DINO (84.3 GFLOPs). These results confirm that ACD-DETR achieves an optimal balance between detection performance and computational efficiency for UAV small-object detection tasks.

The comprehensive cross-paradigm comparison presented in Table 4 demonstrates ACD-DETR’s superior performance on the VisDrone2019 dataset across multiple evaluation metrics. Among end-to-end detection methods, our proposed ACD-DETR achieves a 3.6% improvement in mAP@0.5 (50.9% vs. 47.3%) over the lightweight RT-DETR-r18 baseline while reducing parameter count by 16% (16.3 M vs. 19.88 M). Notably, this performance gain is accomplished with significantly fewer parameters than RT-DETR-r34 (16.3 M vs. 31.1 M) while maintaining comparable computational efficiency (68.9 G vs. 71.3 G FLOPs).

When compared with recent YOLO series models, ACD-DETR maintains substantial performance advantages, outperforming YOLOv11m and YOLOv12m by 7.7% and 9.1% in mAP@0.5, respectively. The model achieves an exceptional balance between precision and recall (63.8% precision and 49.0% recall), demonstrating robust capability in handling the challenging characteristics of aerial imagery, including dense small object detection and complex background scenarios. These results validate that our proposed MSEFM architecture, combined with OG-BC and DPB-AIFI modules, effectively enhances feature extraction, boundary refinement, and spatial modeling for drone-based imagery.

The anchor-free methods show competitive performance, with YOLOv8l achieving 45.8% mAP@0.5 at the cost of higher computational requirements (137.41 G FLOPs). Among anchor-based approaches, Mask-RCNN delivers the strongest results in this category (36.4% mAP@0.5), though this is still significantly behind our method. The performance gap between traditional detectors and end-to-end transformers is particularly evident in precision metrics, where ACD-DETR’s 63.8% surpasses all other methods by at least 2.5 percentage points.

Notably, our model achieves these advancements while maintaining parameter efficiency, with only YOLOv9s and YOLOv10s showing lower parameter counts among all compared methods. These comprehensive results establish ACD-DETR as a new state-of-the-art solution for aerial object detection, particularly in scenarios requiring accurate detection of densely distributed small objects.

**Figure 9:**
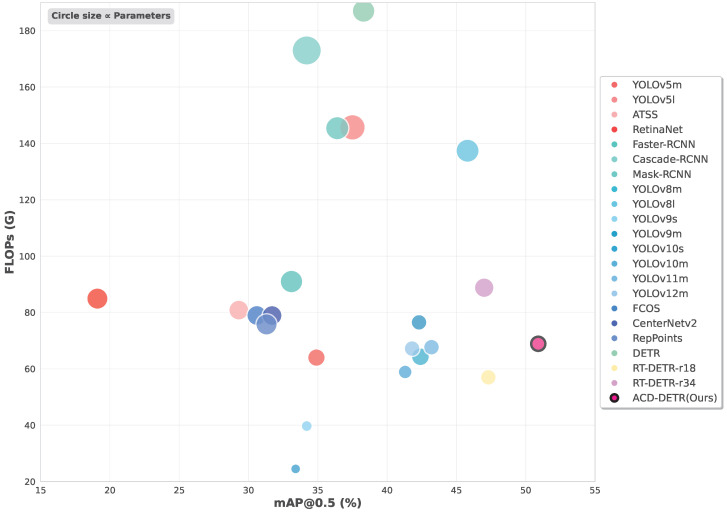
Performance comparison of object detection models: mAP@0.5 vs. computational complexity (FLOPs). The scatter plot shows the trade-off between detection accuracy and computational efficiency across different detection paradigms. Circle size is proportional to model parameters. Four categories are compared: anchor-based one-stage methods (red), anchor-based two-stage methods (cyan), anchor-free methods (blue), and end-to-end methods (green/yellow). Our proposed ACD-DETR (highlighted in pink with black border) achieves competitive accuracy while maintaining relatively low computational complexity and parameter count.

Figure 9 visualizes the accuracy–efficiency trade-off of all compared models. ACD-DETR achieves the highest detection accuracy while maintaining computational complexity below 70 G FLOPs, thus occupying the Pareto-optimal frontier. Compared to lightweight anchor-free models that sacrifice accuracy to reduce computational cost, ACD-DETR maintains a superior precision–recall balance, highlighting its suitability for real-time aerial applications.

Table 5 presents a comprehensive performance comparison across the 10 object categories of the VisDrone2019 dataset, using mAP@0.5 as the evaluation metric. Our proposed ACD-DETR consistently achieves the highest detection accuracy across all categories, demonstrating substantial improvements over the baseline RT-DETR-r18 and other state-of-the-art methods.

Compared to the baseline RT-DETR-r18, ACD-DETR shows significant improvements across all object categories. For challenging small objects, our method achieves notable gains: +3.5% for pedestrian (58.9% vs. 55.4%), +1.9% for people (51.1% vs. 49.2%), and +4.3% for bicycle (26.1% vs. 21.8%). For medium-sized objects such as tricycle and awning-tricycle, ACD-DETR surpasses RT-DETR-r18 by 6.0% and 2.3%, respectively. Even for larger objects like car and bus, consistent improvements of +0.6% and +7.9% are observed, indicating that our proposed modules enhance detection performance across diverse object scales.

Evaluation on the DOTA dataset (Table 6) further validates the effectiveness of ACD-DETR on a different aerial detection benchmark. The model achieves 69.3% mAP@0.5 with strong precision (75.7%) and recall (66.9%), maintaining consistent performance improvements across diverse aerial scenarios. The consistent performance improvements on both the VisDrone2019 and DOTA datasets demonstrate that ACD-DETR’s design is effective across different aerial object detection scenarios, confirming the robustness and broad applicability of our proposed method.

These comprehensive comparisons establish ACD-DETR as a significant advancement in efficient aerial object detection. The consistent improvements across multiple datasets, detection paradigms, and evaluation metrics demonstrate the robustness and practical applicability of our approach for real-world aerial surveillance and monitoring applications.

### 4.5. Ablation Studies

**Figure 10:**
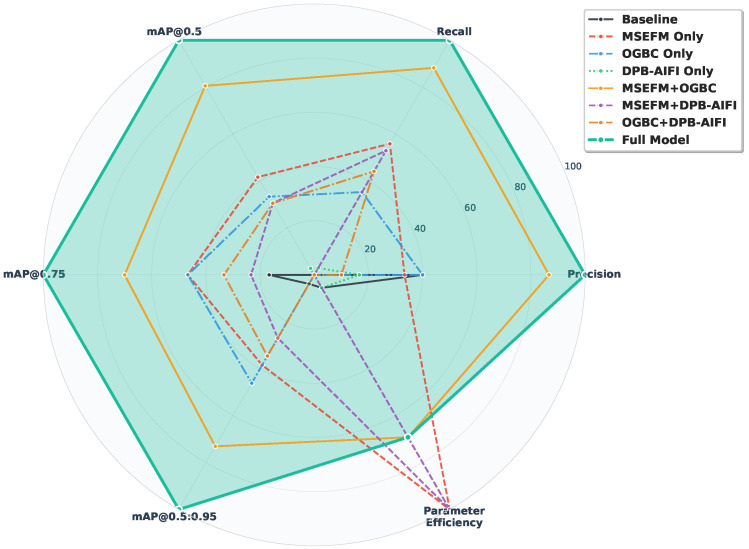
Comprehensive ablation study results on the VisDrone dataset. The radar chart illustrates the normalized performance (0–100 scale) of eight different module combinations across six key evaluation metrics: precision, recall, mAP@0.5, mAP@0.75, mAP@0.5:0.95, and parameter efficiency. Each configuration is represented by a distinct colored line.

To comprehensively evaluate the contributions of our proposed architectural components, we conducted systematic ablation studies examining the individual and combined effects of the MSEFM, OG-BC, and DPB-AIFI modules. The results, presented in Table 7 and visualized in Figure 10, demonstrate the effectiveness of each component across multiple evaluation metrics, including precision, recall, mAP@0.5, mAP@0.75, mAP@0.5:0.95, and parameter efficiency.

Figure 10 presents the comprehensive ablation study results, where MSEFM denotes the Multi-Scale Edge-Enhanced Feature Fusion Module, OG-BC represents the Omni-Grained Boundary Calibrator, and DPB-AIFI indicates the DPB-enhanced AIFI. The radar chart visualization clearly illustrates the progressive performance improvements achieved through different module combinations, with the complete model incorporating all three proposed modules (highlighted with the most prominent line) demonstrating superior performance across all evaluation criteria.

The baseline configuration without any proposed modules achieves 62.0% precision, 45.6% recall, and 47.3% mAP@0.5 with 19.88M parameters. The baseline shows relatively low recall (45.6%) and moderate mAP scores across different IoU thresholds (mAP@0.75: 30.0%, mAP@0.5:0.95: 29.0%). These results highlight the inherent limitations in detecting small objects and achieving precise localization, thus establishing clear motivation for our proposed enhancements. As evident from the radar chart, this baseline configuration forms the innermost performance boundary, serving as a foundation for understanding the individual contributions of each proposed module to the overall system performance.

When integrating MSEFM alone, we observe significant improvements across multiple metrics, as clearly visualized in the radar chart expansion. The mAP@0.5 increases by 1.5 percentage points to 48.8%, while recall improves substantially to 47.5%, demonstrating the module’s effectiveness in enhancing feature extraction capabilities. Notably, the mAP@0.5:0.95 reaches 29.9%, indicating improved performance across varying IoU thresholds. Most remarkably, this enhancement is accompanied by a 26.7% reduction in parameters from 19.88M to 14.57M, demonstrating MSEFM’s dual benefit of improved performance and computational efficiency. The enhanced edge perception and multi-scale feature extraction capabilities of MSEFM prove particularly effective for small object detection scenarios, where precise feature representation is crucial for accurate localization.

The OG-BC module demonstrates comparable effectiveness when applied individually, achieving 48.5% mAP@0.5 and improving recall to 46.8%. The consistent improvements across all mAP metrics (mAP@0.75: 30.9%, mAP@0.5:0.95: 30.1%) validate the module’s capability in enhancing detection robustness through multi-scale semantic fusion and explicit boundary modeling. However, this improvement comes with a slight parameter increase to 20.19M, reflecting the computational cost associated with omni-grained boundary calibration mechanisms. The maintained precision at 62.0% indicates that OG-BC enhances recall without compromising detection accuracy, suggesting effective boundary refinement capabilities.

In contrast, DPB-AIFI alone yields minimal improvements, with mAP@0.5 increasing by only 0.1% to 47.4%, accompanied by slight degradation in precision to 61.3% and mAP@0.5:0.95 to 28.9%. This limited individual contribution, clearly visible as minimal radar chart expansion, suggests that dynamic position bias mechanisms require synergistic interaction with other components to realize their full potential. The minimal improvement indicates that positional encoding enhancements alone are insufficient to address the fundamental challenges in small object detection without complementary feature extraction and boundary modeling improvements.

The combination of MSEFM and OG-BC produces substantial performance gains, achieving 50.2% mAP@0.5, 48.6% recall, and 63.4% precision while maintaining computational efficiency at 16.30M parameters. The 2.9 percentage point improvement in mAP@0.5 compared to the baseline demonstrates strong complementarity between enhanced feature extraction and refined boundary modeling. All mAP metrics show consistent improvements (mAP@0.75: 31.6%, mAP@0.5:0.95: 30.8%), indicating robust performance across varying IoU thresholds. This synergistic effect, prominently displayed in the radar chart, suggests that the multi-scale edge-enhanced features provide a solid foundation for the boundary calibrator to perform more accurate refinements.

In contrast, combinations involving DPB-AIFI without both other modules show limited benefits or slight performance degradation, as evidenced by the constrained radar chart profiles. The MSEFM + DPB-AIFI configuration achieves 48.4% mAP@0.5 with reduced precision (60.8%), while the OG-BC + DPB-AIFI combination maintains similar mAP@0.5 (48.4%) but with lower precision (61.1%). These results suggest that DPB-AIFI’s effectiveness is contingent upon the presence of enhanced feature extraction and boundary modeling capabilities, indicating that dynamic position bias optimization requires a robust feature representation framework to be effective.

The complete ACD-DETR model that incorporates all three modules achieves optimal performance on all evaluation metrics, forming the outermost boundary in the radar chart visualization. The model demonstrates 63.8% precision, 49.0% recall, and 50.9% mAP@0.5 while maintaining computational efficiency with only 16.30M parameters. Compared to the baseline, this represents improvements of 1.8% in precision, 3.4% in recall, and 3.6% in mAP@0.5, with an 18% reduction in parameter count. The consistent improvements across all mAP metrics (mAP@0.75: 32.5%, mAP@0.5:0.95: 31.5%) demonstrate enhanced localization accuracy and robustness across varying detection thresholds. These results confirm that the three architectural components work in concert to maximize detection performance while preserving computational efficiency, with DPB-AIFI providing the final optimization layer that fine-tunes the synergistic interaction between MSEFM and OG-BC. The superior performance of the complete configuration, clearly visible as the most expanded radar chart profile, empirically validates our architectural design principles and confirms the theoretical soundness of the proposed multi-component integration strategy.

### 4.6. Fusion-Enhanced Variant: ACD-DETR-SBA+

Beyond the modular ablation studies, we further propose a fusion-enhanced variant termed ACD-DETR-SBA+, which removes the CSP-OmniKernel module and the DPB-AIFI module, while introducing additional SBA modules to intensify boundary–semantic fusion. This design focuses on enhancing feature-level fusion while discarding explicit positional modeling and omni-kernel calibration.

As shown in Table 8, ACD-DETR-SBA+ achieves superior detection performance across all metrics compared to both the baseline RTDETR-R18 and the full ACD-DETR model. Notably, it outperforms ACD-DETR on mAP@0.5 and mAP@0.5:0.95, demonstrating the effectiveness of repeated semantic–boundary interaction. Although the removal of CSP-OmniKernel and DPB-AIFI reduces the parameter count to 15.67 M, the dense deployment of SBA modules leads to a significant increase in computational complexity, as reflected by the GFLOPs rising to 94.2. This increase is primarily due to the intensive attention mechanisms and repeated fusion operations within the SBA modules.

In summary, ACD-DETR-SBA+ represents a design that sacrifices inference efficiency in favor of improved detection precision. This variant is particularly suitable for scenarios where computational resources are sufficient but detection accuracy is prioritized, such as UAV-based monitoring and inspection tasks.

These results suggest that intensive semantic–boundary fusion, enabled by densely stacked SBA modules, can serve as a lightweight (in terms of parameter count) yet computationally intensive alternative to positional encoding and omni-kernel calibration for UAV small object detection. The ACD-DETR-SBA+ variant highlights the potential of purely feature-level fusion strategies when deployed in resource-rich environments.

### 4.7. Visualization

**Figure 11:**
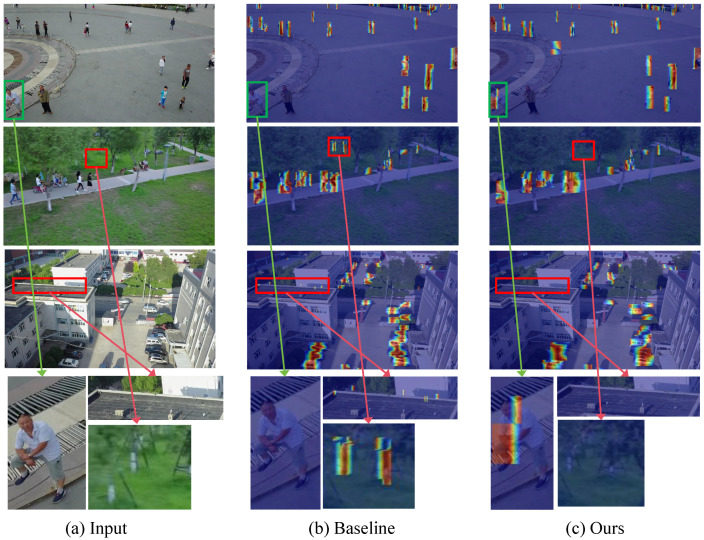
Visual comparison results on the VisDrone dataset. (**a**) Original image. (**b**) Attention heatmap of the baseline model RT-DETR-R18. (**c**) Attention heatmap of the proposed ACD-DETR model. Red boxes indicate false positive detections, and green boxes indicate missed targets.

To qualitatively evaluate the detection performance, we visualize the attention heatmaps of the baseline RT-DETR-R18 and the proposed ACD-DETR on the VisDrone dataset, as shown in Figure 11. Compared to RT-DETR-R18, ACD-DETR exhibits more concentrated and accurate attention on small targets.

As observed from the red and green boxes in Figure 11, the proposed ACD-DETR significantly reduces both false positives and missed detections compared to the baseline model. The attention responses generated by ACD-DETR are more concentrated around actual target regions, leading to more accurate localization. This demonstrates that the integration of multi-scale edge enhancement and spatial-aware encoding improves the model’s capability in detecting small or occluded objects in UAV imagery.

## 5. Conclusions

In this paper, we propose ACD-DETR, a lightweight Transformer-based framework tailored for small object detection in UAV imagery. By integrating MSEFM, OG-BC, and DPB-AIFI, ACD-DETR effectively enhances fine-grained feature extraction, boundary localization, and spatial relationship modeling.

To further investigate the trade-off between detection accuracy and computational cost, we introduce ACD-DETR-SBA+, a fusion-enhanced variant that replaces OG-BC and DPB-AIFI with densely stacked Semantic–Boundary Aggregation (SBA) modules. Although ACD-DETR-SBA+ incurs higher computational overhead (in terms of GFLOPs), it achieves superior detection performance (both mAP@0.5 and mAP@0.5:0.95), making it well-suited for deployment in computation-rich environments.

Extensive experiments on the VisDrone2019 and DOTA datasets demonstrate that ACD-DETR achieves a new state-of-the-art trade-off between accuracy and efficiency for UAV-based small object detection. In contrast, ACD-DETR-SBA+ illustrates the potential of pure feature-level fusion strategies when resources are not constrained. Ablation studies validate the individual and synergistic contributions of each proposed module, while qualitative visualizations show that our model more effectively attends to small and occluded targets than existing baselines.

In future work, we aim to further optimize the fusion strategy, reduce computational complexity, investigate true cross-dataset generalization by evaluating models trained on one aerial dataset directly on other datasets, and adapt the framework for real-time deployment in aerial edge computing scenarios.

## Figures and Tables

**Table 1:** Experimental environment configuration.

Environment Configuration	Name	Version
Hardware	CPU	AMD EPYC 7742 64-Core Processor
	GPU	NVIDIA A100-SXM4-80GB
Software	OS	Ubuntu 22.04.5 LTS
	Python	3.10.17
	CUDA	11.8
	torch	2.3.1

**Table 2:** Core training parameters.

Component	Setting
imgsz	640
batch	4
epochs	200
weight_decay	0.0005
lrf	1.0
lr0	0.0001
momentum	0.9
optimizer	AdamW

**Table 3:** Comparison of recent DETR variants on VisDrone2019.

Method	Backbone	Publication	mAP@0.5 (%)	Params/M	FLOPs (G)
Dynamic-DETR [23]	Swin-T	ICCV 2021	45.2	35.6	85.0
DINO [24]	ResNet-50	ICLR 2023	45.0	38.1	84.3
Drone-DETR [25]	ResNet-50	Sensors 2024	44.1	28.5	68.9
DQ-DETR [26]	ResNet-50	ECCV 2024	44.7	31.0	70.2
ACD-DETR(Ours)	MSEFM	-	50.9	16.2	68.9

**Table 4:** Performance comparison of object detection models on the VisDrone2019 dataset.

Method	Backbone	Publication	Precision/%	Recall/%	mAP@0.5/%	mAP@0.75/%	Params/M	FLOPs (G)
**anchor-based one-stage**								
YOLOv5m	CSP-Darknet53	Ultralytics 2020	47.8	36.8	34.9	24.4	25.0	64.0
YOLOv5l	CSP-Darknet53	Ultralytics 2020	49.8	39.0	37.5	26.1	53.1	145.7
ATSS [27]	ResNet-50	CVPR 2020	42.3	34.2	29.3	20.4	32.17	80.85
RetinaNet [28]	ResNet-50	ICCV 2017	34.5	26.3	19.1	13.3	36.73	84.93
**anchor-based two-stage**								
Faster-RCNN [3]	ResNet-50	NeurIPS 2015	45.5	32.2	33.1	23.2	41.46	90.99
Cascade-RCNN [6]	ResNet-50	CVPR 2018	47.6	33.1	34.2	23.9	69.43	173.0
Mask-RCNN [29]	ResNet-50	ICCV 2017	49.9	35.7	36.4	25.4	43.96	145.41
**anchor-free**								
YOLOv8m	CSP-Darknet53	Ultralytics 2023	54.1	40.6	42.4	29.7	25.8	64.3
YOLOv8l	CSP-Darknet53	Ultralytics 2023	61.3	44.9	45.8	32.2	43.6	137.41
YOLOv9s [9]	G-ELAN	ECCV 2025	47.5	35.4	34.2	23.9	**9.77**	**39.7**
YOLOv9m [9]	G-ELAN	ECCV 2025	55.7	39.9	42.3	26.5	20.02	76.5
YOLOv10s [10]	CSPNet	NeurIPS 2024	45.9	34.3	33.4	23.4	8.06	24.5
YOLOv10m [10]	CSPNet	NeurIPS 2024	53.1	39.7	41.3	25.4	15.32	58.9
YOLOv11m	CSP-ELAN	Ultralytics 2024	55.2	40.8	43.2	26.7	20.03	67.7
YOLOv12m [11]	E-ELAN	arXiv 2025	52.7	41.0	41.8	26.0	20.11	67.2
FCOS [30]	ResNet-50	ICCV 2019	45.1	33.4	30.6	23.4	32.17	78.95
CenterNetv2 [31]	ResNet-50	arXiv 2021	45.6	34.1	31.7	22.3	32.14	78.93
RepPoints [32]	ResNet-50	ICCV 2019	45.2	33.9	31.3	21.9	36.82	75.77
**End-to-End**								
DETR [1]	ResNet-50	ECCV 2020	51.2	39.6	38.3	26.7	41.57	187
RT-DETR-r18 [2]	ResNet-50	CVPR 2024	62.0	45.6	47.3	30.0	19.88	57.0
RT-DETR-r34 [2]	ResNet-50	CVPR 2024	61.3	45.7	47.0	29.5	31.11	88.8
ACD-DETR (Ours)	MSEFM	-	**63.8**	**49.0**	**50.9**	**32.5**	16.30	68.9

**Table 5:** Performance comparison of object detection methods.

Method	YOLOv5m	Mask-RCNN	YOLOv8m	YOLOv9m	YOLOv10m	YOLOv11m	YOLOv12m	RT-DETR-r18	RT-DETR-r34	ACD-DETR
Pedestrian	45.4	39.1	54.7	45.1	43.6	46.8	45.2	55.4	56.1	**58.9**
People	37.3	28.8	47.2	35.7	34.9	36.0	34.7	49.2	48.2	**51.1**
Bicycle	9.4	12.4	19.3	15.0	15.2	17.8	16.9	21.8	20.3	**26.1**
Car	75.0	77.4	85.1	80.6	80.4	81.5	80.9	85.8	85.8	**86.4**
Van	37.1	37.5	49.1	46.4	47.0	48.1	46.9	50.5	51.2	**52.7**
Truck	26.7	34.4	36.8	41.8	39.1	39.9	37.8	37.7	39.3	**41.9**
Tricycle	22.9	25.0	32.1	31.8	29.1	32.8	30.1	33.3	33.5	**39.3**
Awning-tricycle	4.0	11.2	15.7	19.2	16.3	17.8	17.3	18.5	16.0	**20.8**
Bus	42.6	62.4	60.3	60.0	60.8	62.7	61.5	60.4	60.6	**68.3**
Motor	48.3	56.4	58.0	47.8	46.8	48.7	47.3	60.7	59.2	**63.1**
**mAP50**	34.9	36.4	45.8	42.4	41.3	43.2	41.9	47.3	47.0	**50.9**

**Table 6:** Performance comparison of object detection models on the DOTA dataset.

Model	Precision/%	Recall/%	mAP@0.5/%
YOLOv5m	47.2	39.7	42.2
YOLOv5l	68.7	61.2	63.7
ATSS [27]	54.0	46.5	49.0
RetinaNet [28]	68.0	60.5	63.0
Faster-RCNN [3]	67.8	60.3	62.8
Cascade-RCNN [6]	73.3	65.8	68.3
Mask-RCNN [29]	74.0	66.5	69.0
YOLOv8m	73.6	66.1	68.6
YOLOv9m [9]	74.3	64.1	69.1
YOLOv10s [10]	69.9	62.4	64.9
YOLOv10m [10]	72.5	64.5	68.0
YOLOv12m [11]	73.8	64.0	68.9
FCOS [30]	62.6	55.1	57.6
CenterNetv2 [31]	65.2	57.7	60.2
RepPoints [32]	63.2	55.7	58.2
DETR [1]	65.7	58.2	60.7
RT-DETR-r18 [2]	72.8	**67.3**	68.5
ACD-DETR (Ours)	**75.7**	66.9	**69.3**

**Table 7:** Ablation study.

MSEFM	OG-BC	DPB-AIFI	Precision/%	Recall/%	mAP@0.5/%	mAP@0.75/%	mAP@0.5:0.95/%	Params/M
×	×	×	62.0	45.6	47.3	30.0	29.0	19.88
✓	×	×	61.8	47.5	48.8	30.9	29.9	**14.57**
×	✓	×	62.0	46.8	48.5	30.9	30.1	20.19
×	×	✓	61.3	45.7	47.4	29.5	28.9	19.88
✓	✓	×	63.4	48.6	50.2	31.6	30.8	16.30
✓	×	✓	60.8	47.4	48.4	30.2	29.6	**14.57**
×	✓	✓	61.1	47.1	48.4	30.5	29.8	20.19
✓	✓	✓	**63.8**	**49.0**	**50.9**	**32.5**	**31.5**	16.30

**Table 8:** Comparison of ACD-DETR variants and RTDETR-R18 on VisDrone2019.

Model	Precision/%	Recall/%	mAP@0.5/%	mAP@0.75/%	mAP@0.5:0.95	Params/M	FLOPs (G)
RTDETR-R18	62.0	45.6	47.3	30.0	29.0	19.88	60.0
ACD-DETR	**63.8**	49.0	50.9	32.5	31.5	16.30	68.9
ACD-DETR-SBA+	62.7	**50.5**	**52.0**	**34.7**	**33.1**	**15.67**	**94.2**

## Data Availability

The raw data supporting the conclusions of this article will be made available by the authors upon request.
